# The Role of Digital Technology in Preventing Anemia Among Pregnant Women: A Scoping Review

**DOI:** 10.1155/ijta/5541886

**Published:** 2025-10-10

**Authors:** Husnul Khotimah, Tris Eryando, Agung Dwi Laksono, Ray Wagiu Basrowi

**Affiliations:** ^1^Doctoral Program in Public Health Sciences, Faculty of Public Health, Universitas Indonesia, Depok, Indonesia; ^2^Faculty of Health Sciences, Universitas Faletehan, Serang, Indonesia; ^3^National Research and Innovation Agency Republic of Indonesia, Jakarta, Indonesia; ^4^Indonesia Health Development Center, Jakarta, Indonesia; ^5^Department of Community Medicine, Faculty of Medicine, Universitas Indonesia, Jakarta, Indonesia

**Keywords:** accuracy, anemia, machine learning, prediction, pregnant women

## Abstract

**Objective:**

Anemia remains a major public health concern among pregnant women, significantly contributing to maternal and infant morbidity and mortality. With advances in digital technology, particularly machine learning (ML), there is potential to enhance early detection and prevention strategies. This scoping review is aimed at synthesizing the existing literature on the application of ML-based digital tools for predicting and preventing anemia in pregnant women.

**Methods:**

Following the PRISMA-ScR guidelines, this review included articles from PubMed, Scopus, and Google Scholar published between 2015 and 2024. Eligibility criteria included studies using ML methods for anemia prediction in pregnant women. Extracted data included study population, ML algorithms used, and performance metrics. Due to the scoping nature of this review, risk of bias assessment was not conducted.

**Results:**

A total of 11 studies met the inclusion criteria, utilizing various ML algorithms such as decision trees, random forest, support vector machines, naive Bayes, *K*-nearest neighbors, PART, fuzzy Tsukamoto, and boosting algorithms. Comparative analysis based on performance metrics (accuracy, precision, recall, *F*1-score, and AUC) identified boosting and random forest as the most effective models in resource-limited settings. However, heterogeneity in data sources, evaluation metrics, and populations limits generalizability.

**Conclusions:**

ML-based models demonstrate considerable promise in predicting anemia among pregnant women. However, further validation across diverse datasets, clearer articulation of methodological strengths and limitations, and attention to implementation feasibility in low-resource settings are needed.

## 1. Introduction

Anemia remains a significant public health concern during pregnancy due to its association with increased risks of maternal, fetal, and neonatal mortality. According to estimates by the World Health Organization (WHO) in 2019, approximately 37% of pregnant women globally and 40% of children under the age of five are affected by anemia. The prevalence is particularly high in West African, Middle Eastern, and South Asian countries [1].

In Indonesia, the prevalence of anemia among pregnant women was 37.1% in 2013 [2] and escalated to 48.9% by 2018, surpassing the global rate among pregnant women [3]. However, based on the 2023 Indonesian Health Survey, this figure declined to 27.7%. This reduction is particularly notable when compared to the 2018 Basic Health Survey results. The most significant decline was observed among pregnant women aged 15–24 years (from 84.6% to 14.5%), whereas in the 25–34 age group—commonly associated with pregnancies—the decrease was only 2.3% (from 33.7% to 31.4%). Although the iron supplementation program in Indonesia reached 92.2% coverage, only 44.2% of pregnant women consumed iron supplements as recommended. Low compliance remains a major factor contributing to the high rate of anemia during pregnancy [4].

To prevent maternal anemia, it is necessary first to identify the causes and risk factors. Iron deficiency is the primary cause, accounting for approximately half of all cases, though other etiologies may also contribute [5–7]. A literature review indicates that the risk factors for anemia in pregnant women can be mapped using the epidemiological triangle model. This model provides a framework for understanding the interaction among three key elements: host, agent, and environment. “Host” factors refer to intrinsic human characteristics that can initiate or influence disease progression. The “agent” is a substance whose presence or absence can cause disease. “Environmental” factors refer to external conditions that enable disease emergence [8–10].

These risk factors can serve as variables in predicting anemia during pregnancy. Given the high prevalence of anemia, technological innovations hold substantial potential. Digital interventions can enhance diagnostic capabilities and support the development of effective treatment strategies [11]. Digital technology, particularly artificial intelligence (AI), has emerged as a promising approach in anemia prevention. AI-based tools, such as those utilizing machine learning (ML), can model relationships between anemia-related variables and outcomes such as anemia severity [11–13]. The employment of ML trained on big data can yield more accurate predictive models [14].

ML models are capable of diagnosing and forecasting a variety of illnesses and health issues through pattern recognition, visualization, trend analysis, and regularity identification in data [15–19]. The foundation of ML is the notion that a system can learn from data, identifying important patterns for improved decision-making with less assistance from humans. ML algorithms have demonstrated efficacy in the early and more accurate prediction of fatal diseases, including anemia, hepatitis, lung cancer, liver disease, breast cancer, thyroid illness, and diabetes. These algorithms are instrumental in analyzing healthcare data to facilitate disease diagnosis, improve quality of life, prevent premature deaths, and predict epidemics. In essence, ML plays an important role in health informatics [20]. Digital technology facilitates the rapid and precise evaluation of vast volumes of data, aiding in the prevention of anemia in pregnant women [21].

ML has emerged as a useful instrument for deciphering intricate data sets and identifying trends in a variety of sectors, including healthcare. In predicting health data, the effectiveness of random forest (RF), extreme gradient boosting (XGBoost), logistic regression (LR), artificial neural network (ANN) models, decision tree (DT), ensemble learning, *k*-nearest neighbor (KNNN), and naïve Bayes has been demonstrated [22–25].

Anemia disease analysis involves the application of learning-based classification techniques to model the relationship between input factors pertaining to anemia conditions and goal variables, which may include the degree of anemia or other relevant parameters. Classification, characterized by its simplicity and the clarity it provides regarding the relationships between variables, is frequently used in this context. Classification is the process of organizing objects according to the traits they possess in common. Various methodologies exist for classifying the procedure, including the use of technology or manual methods. Manual classification involves the use of human expertise to categorize data without the aid of sophisticated computer algorithms. Conversely, classification that is performed with the help of technology employs various algorithms, including naïve Bayes, support vector machine (SVM), DT, fuzzy, and ANNs [26]. The objective of this study is to examine the efficacy of digital technologies, particularly ML methods, in preventing anemia among pregnant women.

## 2. Methods

### 2.1. Study Design

This scoping review was conducted through a structured literature search of studies published up until December 2024 and was used to perform this scoping review. The review was conducted and reported in accordance with the Preferred Reporting Items for Systematic Reviews and Meta-Analyses (PRISMA) guidelines [27], which offered an organized framework. The PICOS framework was used to find pertinent studies. The population, intervention, comparator, outcomes and study design make up this framework. Following the identification of potentially pertinent research, all identified articles were imported into a reference management software to detect and eliminate duplicate entries, guaranteeing a clean dataset for additional examination.

This scoping review was prospectively registered in the Open Science Framework (OSF) under the identifier https://osf.io/xar7m. The protocol outlined the objectives, inclusion criteria, and methods in accordance with the JBI Manual for Evidence Synthesis and the PRISMA-ScR checklist. During the review process, a minor modification was made to the eligibility criteria, specifically the inclusion of studies published from 2010 onwards instead of 2015, to ensure the capture of earlier ML applications relevant to anemia prediction. All methodological deviations from the original protocol were documented and justified accordingly. This additional transparency ensures that distinctions are made between preplanned decisions and those adapted during the review process.

### 2.2. Search Strategy

A comprehensive search was conducted of articles that were released between 2015 and 2024 in the PubMed, Scopus, and Google Scholar databases. Similar adaptations were made to accommodate the unique tagging systems for Scopus and Google Scholar, using relevant field tags and filters. The last search was conducted on December 14, 2024. A complete and reproducible search strategy is now provided in the supporting material to ensure transparency and facilitate future replication. While the inclusion criteria specified English-language studies, several relevant articles originally published in Bahasa Indonesia were included due to their scientific value and availability of English translations or bilingual abstracts. Full texts in Bahasa Indonesia were reviewed by bilingual authors to ensure accurate interpretation. Regarding the risk of bias, no formal risk of bias assessment was conducted, consistent with JBI guidance for scoping reviews. However, the absence of such assessment is acknowledged as a limitation, and readers are cautioned that variations in study quality may influence the reliability of the synthesized findings. The source search strategy is outlined in Table 1.

### 2.3. Eligibility Criteria

The following included criteria were established for the selected articles: First, the articles must discuss the risk factors for anemia in pregnant women; second, the articles must employ digital technology methods in predicting anemia in pregnant women using ML approaches; and third, the articles must be in the form of journals, proceedings, full text, and open access. Articles pertaining to the utilization of digital technology in contexts other than anemia in pregnant women, short articles, case reports, letters to the editor, review articles, and other reports were not included in the study.

### 2.4. Study Selection

**Figure 1:**
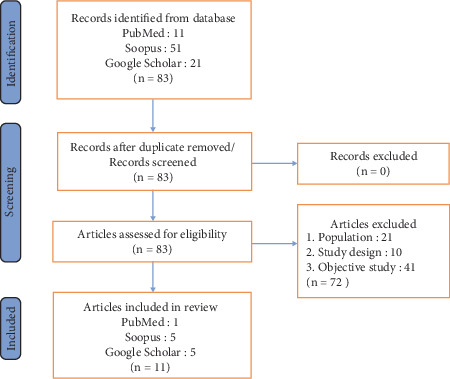
Visual representation of the selection process for related article.

To reduce bias, the article selection procedure was rigorous and multiphase. All recognized articles' titles and abstracts were first checked for relevancy, and those that did not fit the inclusion requirements were eliminated using Mendeley Reference Manager. Articles that passed this preliminary screening were subjected to a full-text examination to verify their eligibility. Any disagreements or ambiguities were addressed during this process by the reviewers themselves, and they consulted with a third reviewer where required to arrive at a consensus. Only pertinent and excellent papers were included in the evaluation due to this stringent selection procedure. Creating a thorough summary of the research's features and doing an analysis of articles related to the theme of the results presented in Microsoft Office Excel 2016 were the two main components of the synthesis. Discrepancies in coding and interpretation were resolved through consensus discussion. Then, 84 records were found during the scoping review's search and selection procedure, from PubMed (*n* = 11), Scopus (*n* = 51), and Google Scholar (*n* = 21). After the initial screening, 83 records were retained, 72 of which were excluded for various reasons, including unsuitable study design, demographic factors, and lack of relevance to the study's goal. The final number of studies that were retrieved was 11, and all of these were successfully assessed for eligibility. The visual representation of the selection process for related articles is outlined in Figure 1.

### 2.5. Data Extraction

Data were extracted using a structured form with five main components: (1) the title of the article and its bibliographic details, journal name, author name, year of publication of the article, and location of the study; (2) research objectives and variables studied; (3) research methods; (4) data or samples used; and (5) research results. Subsequent to the data collection, a content analysis technique was conducted to systematically analyze, summarize, and report the data in accordance with the study's objectives.

## 3. Results

### 3.1. Characteristics of the Included Studies

A total of 11 articles were reviewed, focusing on the population, variables, and methods. In this case, the primary focus was on the utilization of digital technology in the prevention of anemia in pregnancy. The articles identified employed a ML approach for predicting anemia. A systematic determination of categories or limitations was conducted for each article, encompassing the following aspects: (1) The population was pregnant women; (2) the objective was prediction of anemia in pregnant women or the presence of variables about anemia in pregnant women as both dependent and independent variables; (3) the intervention was in the form of the use of a ML approach for data analysis; and (4) the outcome was the accuracy, precision, recall, *F*1-score, and area under the curve (AUC) value of the algorithm's data results. The result of data extraction is described in Table 2.

### 3.2. Types of ML Methods

**Figure 2:**
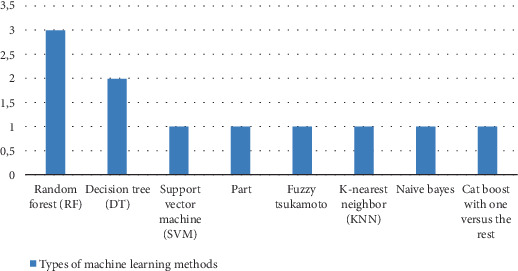
Types of machine learning methods.

Figure 2 illustrates the distribution of ML methods used across the reviewed studies for predicting anemia in pregnant women. Among the algorithms analyzed, RF appeared most frequently (in three studies), highlighting its growing popularity in handling large datasets, reducing overfitting, and delivering robust classification performance.

DT methods were the second most commonly used, appearing in two studies, likely due to their simplicity, interpretability, and effectiveness in modeling categorical variables related to anemia risk.

Other algorithms—including SVM, PART, Fuzzy Tsukamoto, KNN, Naïve Bayes, and CatBoost with one-versus-rest—were each used in a single study. This indicates exploratory use of a diverse range of ML techniques, but with limited comparative evidence due to their singular appearance. The variety of methods reflects ongoing experimentation in the field and a lack of consensus on a universally superior algorithm for this specific application.

### 3.3. Outcome Performance Metrics

**Figure 3:**
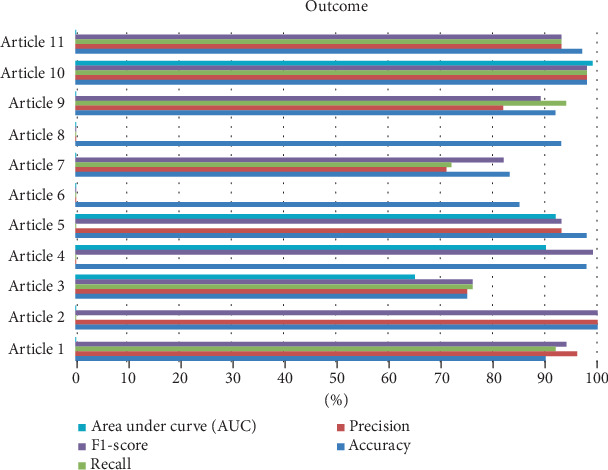
Outcome performance metrics.

Figure 3 presents the performance outcomes of ML models across 11 articles using five key evaluation metrics: accuracy, precision, recall, *F*1-score, and AUC. Most studies reported high accuracy, with Articles 1, 2, 5, 10, and 11 approaching or exceeding 95%, indicating strong overall predictive capabilities. Notably, Article 11 demonstrates the most consistent and balanced performance across all metrics, suggesting a well-generalized model with high discriminatory power.

Precision and recall varied more substantially across studies. For instance, Article 7 showed relatively lower values for recall and *F*1-score, despite high precision, implying potential bias toward correctly identifying only positive cases and possibly overlooking negative ones. The AUC values were explicitly reported in only a few studies, with Articles 5 and 11 displaying AUCs near or above 90%, reflecting excellent classification performance.

The observed discrepancies between accuracy and other metrics such as recall and *F*1-score (e.g., in Articles 3 and 6) highlight the importance of evaluating models holistically rather than relying on a single metric. In clinical contexts like anemia prediction, recall, and *F*1-score are especially important to minimize false negatives and ensure at-risk individuals are identified correctly.

## 4. Discussion

ML, a subfield of AI, focuses on deriving insights from data. The goal of ML is to develop systems that can learn “independently,” without the need for constant human programming. ML employs a variety of models, including naïve Bayes, KNNs, SVMs, DTs, and LR. The terms “supervised learning” and “unsupervised learning” are also used in the context of machine learning. Supervised learning, which relies on a teacher or supervisor to classify training examples into classes and use information about each training example's class membership, is achieved through trial-and-error interactions with its environment (reward/penalty assignment). In contrast, unsupervised learning uses heuristics to identify patterns in class material. There are two types of ML models: ensemble learning and single model. Ensemble learning, being a supervised learning technique, is predicated on the premise that it can be learned and used to generate predictions. This learning model combines the applications of the single model utilized, thereby offering a high degree of versatility by combining one or more single model methods [33–35].

Recent advances in maternal healthcare technology and ML offer promising frameworks for early anemia detection. Studies such as [36, 37] have demonstrated efficient data routing and fusion systems that can potentially enhance maternal health monitoring through real-time, wearable body area networks and integrated AI systems. These approaches could be adapted to anemia surveillance in pregnancy, particularly in rural or low-resource areas where real-time diagnostics are not always available. Similarly, [38] highlighted the use of hybrid predictive analytics in neurological healthcare, which may inspire more sophisticated, multi-modal approaches for maternal anemia risk prediction using machine learning.

Beyond the algorithmic focus, improving maternal anemia outcomes also requires attention to upstream interventions. A recent systematic review by [39] confirmed that preconception nutritional supplementation significantly reduces anemia and supports intrauterine growth. These findings suggest that ML-based risk screening should be paired with community-level nutritional strategies to maximize clinical benefit.

One of the most important tools for managing unstructured free text data is machine learning. The employment of both supervised and unsupervised methods is contingent upon the data source. These methods facilitate healthcare organizations in gaining knowledge from the enormous volumes of unstructured free-text data, a task that is further facilitated by the continuous advancement of data analysis tools [40].

The literature review of 11 selected articles revealed the efficacy of ML methods in predicting anemia in pregnant women. The study identified the DT, RF, SVM, part, fuzzy Tsukamoto, KNN, naïve Bayes, and boosting algorithms with one-versus-rest as the most accurate methods. The subsequent sections will present a discussion of how each of these ML methods can prevent anemia in pregnancy by predicting the factors that influence it.

### 4.1. DT

A method for visualizing the decision-making process is to create a diagram that illustrates the array of possible choices and their respective impacts. This approach enables individuals to contemplate an extensive range of potential actions. The diagram, often referred to as a DT, usually commences with a single node that bifurcates into multiple branches, each representing a distinct choice. Each subsequent branch then has its own branches [41]. A specific ML technique known as a DT classifier separates data into subsets according to the values of input attributes. This method produces a DT model, where each leaf node indicates the outcome or class name, each internal node represents a feature, and each branch indicates a decision rule [21].

In the domain of machine learning, DT classifiers are classified as supervised learning, defined as a modeling technique where the data to be examined contains labels or classifications. The primary objective is to understand the causal relationship between the independent variable and the dependent variable, which are often known as the target or label in this context [11].

A study by Susanti (2024) demonstrated that the DT is the most effective classification model for predicting anemia on the utilized dataset. The evaluation of the model using various metrics, such as accuracy, precision, recall, and *F*1-score, provides a comprehensive assessment of its capability to classify and predict anemia. The findings of the study demonstrated that the DT model exhibited consistent and superior performance in predicting anemia, achieving accuracy, precision, recall, and *F*1-score values of 1.0 on all tested data ratios [11, 25].

In this review, the explanation of ML algorithms has been refocused to emphasize their practical application in predicting anemia among pregnant women, rather than relying on general textbook definitions. DT algorithms have shown particular utility in modeling categorical variables relevant to anemia risk, such as iron supplement intake, dietary habits, or maternal age. Their interpretability enables healthcare practitioners to understand the rationale behind each classification, which is critical in clinical decision-making [11].

### 4.2. RF

RF is an algorithm used for the classification of voluminous datasets. To enhance the precision of predictions, this algorithm constructs multiple DTs and integrates them. Bagging methods, which amalgamate learning models to improve the overall performance, are usually used to train these sets of trees [41]. To increase accuracy and generalization, RF, an ensemble learning system, aggregates multiple weak classifiers and selects or averages the final results [42] .The RF algorithm is a notable example of such a strategy, demonstrating resilience against overfitting and the capacity to effectively handle a high volume of input features. The RF algorithm constructs individual trees through the use of feature randomization and bagging, thereby yielding a forest of uncorrelated trees. The collective prediction accuracy of this forest surpasses that of any individual tree. The algorithm's design aims to enhance the accuracy of successive models by combining both weak and strong learners, thereby creating a more robust and accurate predictive system [43].

Anemia during pregnancy poses a grave threat to public health, especially in environments with limited resources. ML holds considerable promise for enhancing anemia identification and treatment. A study conducted in Ethiopia revealed that the RF classifier demonstrated optimal performance across all categories, exhibiting 97% accuracy, 93% precision, 93% recall, and 93% *F*1-score. This study also noted low false positive rates and high true positive rates. The results of previous research conducted in Ethiopia found that a more comprehensive understanding of the risk factors for anemia and the benefits of the RF method in predicting anemia in pregnant women is needed so that early detection and appropriate intervention can be carried out [21].

RF, as an ensemble learning method, is widely employed due to its robustness in handling high-dimensional data and minimizing overfitting. In the context of maternal anemia prediction, RF models have been successfully used to process large datasets such as demographic health surveys, effectively capturing complex interactions among sociodemographic and biomedical factors. Its ability to rank feature importance also aids in identifying key risk factors contributing to anemia in pregnancy [21].

### 4.3. SVM

SVM is a supervised learning method used in regression and classification. The SVM concept is mathematically advanced in comparison to other classification methods. The SVM maximizes the distance between classes to identify the optimal hyperplane. SVMs can be categorized into two types: linear and nonlinear. Linear SVMs are used for linearly separable data [41]. Notwithstanding the evidence from certain studies indicating the SVM's inferior performance in comparison to other methods, the findings from its application in a study focused on anemia prediction in Bangladesh demonstrated a considerable degree of accuracy [44]. The SVM is often used as a comparative approach to identify the most suitable method for predicting health problems in several studies. In a study by Tamir et al., 2017 [45], the SVM technique achieved an accuracy of 78.9% in detecting anemia through the analysis of photographs of the conjunctiva of the eye [46].

SVM has been used in more specialized applications, particularly when integrating biometric data or clinical imaging. One notable study applied SVM to analyze conjunctival images for anemia detection, highlighting its effectiveness in binary classification tasks and its potential in non-invasive screening tools. While SVM may offer high classification accuracy, its relative lack of transparency compared to DT or RF may pose challenges for implementation in routine maternal care, where explainability is essential [45].

### 4.4. PART

The PART method is a relatively simple algorithm that does not use global optimization to derive precise rules. Rather, it generates partial DTs and retrieves rules one at a time. The algorithm repeatedly generates a recursive rule for instances until no further examples remain after removing instances covered by a rule [28]. Another supervised ML technique, the PART algorithm, identified 19 rules. The PART algorithm posits that the prevalence of anemia is lower in mothers under 34 years old, those who take iron supplements, and those who drink less tea [47–51]. The most effective preventive factor against anemia is pregnancy-related iron supplementation [52].

According to the findings of a study by Kaya et al., the increased incidence of anemia is associated with the consumption of dark tea with meals, multigravida, advanced maternal age, advanced gestational age, inadequate iron support, and dietary diversity. The accuracy of three distinct algorithms developed for the study ranged from 85.5% to 97.9% in predicting anemia in pregnant women. However, the translation of these findings into a clinical decision support system and their implementation in daily practice require further study [28].

### 4.5. Fuzzy Tsukamoto

Anemia, characterized by low hemoglobin levels in the human body, can manifest with a variety of symptoms, including fatigue, weakness, and lightheadedness. This condition can impair cognitive function, physical stamina, and resistance to illness. Early detection, informed by symptoms, is crucial for the accurate diagnosis of anemia. A system using the fuzzy Tsukamoto algorithm can compute anemia symptom values. This system allows users to input the values of symptoms they have encountered, such as hemoglobin levels, bleeding, and weakness. During the fuzzification stage, the input symptom value is converted to a fuzzy value ranging from 0 to 1. In the rule construction stage, 18 rules are derived from three symptoms and three diagnosis outcomes. Subsequent to obtaining the rule, the inference engine uses the min function to obtain the *α*-predicate value in each rule. Finally, defuzzification is performed to obtain the output value or cris*p* value after obtaining the *α*-predicate value. The accuracy value of the prediction data and the data derived from the fuzzy Tsukamoto algorithm is 85% when using the multiple confusion matrix method. Through the website, the general public can use this technique to identify anemia earlier [29].

### 4.6. KNN

The KNN algorithm is a learning method that categorizes data based on the similarity between inputs. The geometric distance method, which is determined by the number of independent variables, can be used to calculate the distance between two points in the KNN algorithm [41]. A study conducted in Bangladesh utilized the KNN in conjunction with two additional techniques, SVM and DT, to predict anemia. The data for this study were gathered from hemoglobin tests, anemic conditions, and palpebral conjunctiva images, the latter of which were captured using a high-resolution cell phone camera. The image processing method in MATLAB was then used to extract the percentage of red, green, and blue pixels from the images. Finally, the hemoglobin level was plotted using these features. The dataset for the study comprised 23 data points for testing and 81 data points for training. The results of the study indicated that the KNN algorithm, despite not attaining the highest level of accuracy, can serve as a reliable predictor of anemia [53].

### 4.7. Naïve Bayes

*Naïve Bayes* is a classifier that utilizes conditional probabilities to assign class labels to examples, thereby operating as a supervised, object-based classification technique [41]. The utilization of a ML algorithm to develop an early detection of anemia in patients with low hemoglobin (Hb) levels and to identify which parameters have the biggest impact has been explained in a paper that found that the naïve Bayes algorithm achieved a 99.96% accuracy in detecting anemia. So, this method can be used to predict anemia in pregnant women [54].

### 4.8. Boosting With One-Versus-Rest

The boosting approach introduced by Freund and Schapire has emerged as a potent tool for binary class classifiers [55, 56]. In a study by [32], the CatBoost algorithm was selected over other algorithms for future applications in risk factor analysis, model deployment, artifact development, and rule generation. This selection was based on the algorithm's demonstrated superior performance. In addition, the study identified the most significant risk variables for anemia in pregnant women, as determined by feature importance. The determination of the best-performing algorithms' determinant risk variables was made through a feature importance analysis.

### 4.9. Comparative Interpretation and Research Implications in Predicting Anemia in Pregnant Women

A subfield of AI known as ML is concerned with the development of algorithms that enable systems to learn from data and improve performance over time without being explicitly programmed [57]. The goal is to build predictive models capable of recognizing patterns and making decisions autonomously, making ML particularly valuable in health informatics and medical diagnostics [58]. This review offers a structured synthesis of ML applications for anemia prediction in pregnant women, organized by algorithm type—such as DT, RF, SVM, and boosting. This classification enables researchers to evaluate methodological suitability based on data complexity, availability, and implementation context [59]. Each method is discussed in terms of performance, data requirements, interpretability, and scalability, thereby serving as a comparative reference for those developing or refining ML tools in maternal health, especially within resource-limited environments [60].

To enhance result interpretation and engagement, we recommend visual formats such as heatmaps, bar plots for model performance, and bubble plots comparing accuracy to population size. Such data visualizations improve comprehension of findings and support informed decision-making for public health practitioners and policymakers. Methodological descriptions have been contextualized within anemia prediction frameworks. For instance, DTs have been effectively used to model categorical inputs such as dietary intake and supplement adherence [11], while RFs accommodate large, heterogeneous health datasets with nonlinear relationships [21]. SVMs, although less interpretable, have shown promise when applied to biometric and imaging data relevant to maternal anemia risk [45].

Despite encouraging accuracy metrics, several research gaps remain. Most models are trained using localized datasets without external validation, limiting generalizability. Ensemble methods such as boosting and RF consistently outperform simpler models in predictive power, but may be too resource-intensive for deployment in primary care or rural settings [20]. Simpler approaches like LR or single-tree models could offer acceptable trade-offs between accuracy and feasibility. Future research must address these gaps through multicenter validation, attention to deployment cost, and usability testing in real-world maternal health contexts.

### 4.10. Study Limitation

This review has several limitations. First, heterogeneity across studies in terms of datasets, population demographics, and ML implementation makes direct comparison challenging. Second, evaluation metrics varied widely among studies, with some reporting only accuracy while others included precision, recall, or *F*1-score, impeding consistent cross-study assessment. Third, no formal quality or risk of bias appraisal was conducted due to the scoping nature of the review, which may affect the reliability of aggregated insights. Lastly, language restrictions and inclusion of translated non-English sources may introduce interpretive biases.

## 5. Conclusion

This scoping review systematically identified eight ML algorithms that have been applied to predict anemia in pregnant women. Among these, the boosting algorithm and RF consistently demonstrated the highest overall performance in terms of accuracy and reliability. These findings highlight the promising role of ML in supporting early, noninvasive, and cost-effective anemia prevention strategies, particularly in resource-limited settings where traditional diagnostic tools may be inaccessible or delayed.

However, the integration of these ML tools into clinical or community-based healthcare systems requires careful attention to several critical aspects. These include rigorous external validation using diverse datasets, the development of standardized evaluation frameworks, and a thorough assessment of practical implementation barriers such as infrastructure, data availability, and end-user readiness.

This review provides a comprehensive synthesis of current ML applications in the field of maternal health. Moreover, it identifies key methodological gaps and proposes future directions for research and practice. The insights presented in this review are intended to support researchers, clinicians, and public health practitioners in selecting, adapting, and optimizing ML approaches tailored to the prediction and prevention of anemia in pregnant populations.

## Figures and Tables

**Table 1:** Sourcing strategy.

**Year limitation**	**Language**	**Database sources**	**Keyword**
2015–2024	English	PubMed	(“anemia”[MeSH Terms] OR “anemia”[All Fields]) AND (“pregnancy”[MeSH Terms] OR “pregnant women”[All Fields]) AND (“machine learning”[MeSH Terms] OR “machine learning”[All Fields]) AND (“prediction”[All Fields] OR “prevention”
	English	Scopus	
	English	Google Scholar	

**Table 2:** Summary of machine learning algorithms and performance for anemia prediction.

**Author/year/country**	**Population**	**Objective**	**ML algorithm**	**Outcome performance metrics**				
				**Accuracy**	**Precision**	**Recall**	**F**1** -score**	**Area under curve (AUC)**
Munawwaroh and Primandari, 2022, Indonesia	147 pregnant women (train-test split 80:20)	Classifying mid-upper arm circumference (MUAC)	Decision tree (DT)	90%	96%	92%	94%	—
Susanti & Elfianti, 2024, Indonesia	Kaggle dataset (422 rows, 6 features)	Anemia prediction	Decision tree (DT)	100%	100%	—	100%	—
Harahap *et al.,* 2024, Indonesia	1280 pregnant women attending antenatal care	Prediction of neonatal health status	Support vector machine (SVM)	75%	75%	76%	76%	65%
Adebanji, Asare, and Gyamerah, 2024, Africa	6466 women with premature births (< 32 weeks gestation)	High-risk pregnancy prediction	Random forest (RF)	98%	—	—	99%	90%
Kaya et al., 2023, Turkey [28]	495 pregnant women	Predicting iron deficiency anemia using sociodemographic and clinical factors	PART	97.98%	92.67%	—	92.67%	91.67%
Ningrum et al., 2021, Indonesia [29]	40 pregnant women	Anemia prediction	Fuzzy Tsukamoto	85%	—	—	—	—
Sinambela et al., 2023, Indonesia	3258 pregnant women	Postpartum bleeding prediction	Random forest (RF)	83%	71%	72%	82%	—
Triana et al., 2023, Indonesia [30]	45 pregnant women	High-risk pregnancy detection	*K*-nearest neighbor (KNN)	93%	—	—	—	—
Pusadan et al., 2023, Indonesia [31]	600 pregnant women	Optimal delivery process prediction	*Naïve Bayes*	92%	82.4%	94%	88.7%	—
Dejene et al., 2022, Ethiopia [32]	29,104 instances from the Ethiopian Demographic Health Survey (EDHS)	Predicting anemia rates in pregnant women	CatBoost (one-vs.-rest)	97.6%	97.6%	97.6%	97.6%	99%
Kitaw et al., 2024, Ethiopia [21]	11,174 instances from the Ethiopian Demographic Health Survey (EDHS)	Anemia severity prediction among ANC attendees	Random forest (RF)	97%	93%	93%	93%	—

## Data Availability

All data used to support the results of this study are included in the article.
